# Evaluation of a Novel Adjuvanted Vaccine for Ultrashort Regimen Therapy of *Artemisia* Pollen-Induced Allergic Bronchial Asthma in a Mouse Model

**DOI:** 10.3389/fimmu.2022.828690

**Published:** 2022-03-15

**Authors:** Kairat Tabynov, Meruert Babayeva, Tair Nurpeisov, Gleb Fomin, Temirzhan Nurpeisov, Ulbossyn Saltabayeva, Sankar Renu, Gourapura J. Renukaradhya, Nikolai Petrovsky, Kaissar Tabynov

**Affiliations:** ^1^ International Center for Vaccinology, Kazakh National Agrarian Research University (KazNARU), Almaty, Kazakhstan; ^2^ Preclinical Research Laboratory With Vivarium, M. Aikimbayev National Research Center for Especially Dangerous Infections, Almaty, Kazakhstan; ^3^ T&TvaX LLC, Almaty, Kazakhstan; ^4^ Department of General Immunology, Asfendiyarov Kazakh National Medical University (KazNMU), Almaty, Kazakhstan; ^5^ Republican Allergy Center, Research Institute of Cardiology and Internal Medicine, Almaty, Kazakhstan; ^6^ Nursing Department, Astana Medical University, Nur-Sultan, Kazakhstan; ^7^ Center for Food Animal Health, Ohio Agricultural Research and Development Center, The Ohio State University (OSU), Wooster, OH, United States; ^8^ Vaxine Pty. Ltd., Flinders University, South Australia, Australia

**Keywords:** allergy, vaccine, allergen-specific immunotherapy, wormwood pollen, Art v 1 protein, adjuvant, mice

## Abstract

Wormwood (*Artemisia*) pollen is among the top 10 aeroallergens globally that cause allergic rhinitis and bronchial asthma. Allergen-specific immunotherapy (ASIT) is the gold standard for treating patients with allergic rhinitis, conjunctivitis, and asthma. A significant disadvantage of today’s ASIT methods is the long duration of therapy and multiplicity of allergen administrations. The goal of this study was to undertake a pilot study in mice of a novel ultrashort vaccine immunotherapy regimen incorporating various adjuvants to assess its ability to treat allergic bronchial asthma caused by wormwood pollen.

We evaluated in a mouse model of wormwood pollen allergy candidates comprising recombinant Art v 1 wormwood pollen protein formulated with either newer (Advax, Advax-CpG, ISA-51) or more traditional [aluminum hydroxide, squalene water emulsion (SWE)] adjuvants administered by the intramuscular or subcutaneous route vs. intranasal administration of a mucosal vaccine formulation using chitosan-mannose nanoparticle entrapped with Art v 1 protein. The vaccine formulations were administered to previously wormwood pollen-sensitized animals, four times at weekly intervals. Desensitization was determined by measuring decreases in immunoglobulin E (IgE), cellular immunity, ear swelling test, and pathological changes in the lungs of animals after aeroallergen challenge. Art v 1 protein formulation with Advax, Advax-CpG, SWE, or ISA-51 adjuvants induced a significant decrease in both total and Art v 1-specific IgE with a concurrent increase in Art v 1-specific IgG compared to the positive control group. There was a shift in T-cell cytokine secretion toward a Th1 (Advax-CpG, ISA-51, and Advax) or a balanced Th1/Th2 (SWE) pattern. Protection against lung inflammatory reaction after challenge was seen with ISA-51, Advax, and SWE Art v 1 formulations. Overall, the ISA-51-adjuvanted vaccine group induced the largest reduction of allergic ear swelling and protection against type 2 and non-type 2 lung inflammation in challenged animals. This pilot study shows the potential to develop an ultrashort ASIT regimen for wormwood pollen-induced bronchial asthma using appropriately adjuvanted recombinant Art v 1 protein. The data support further preclinical studies with the ultimate goal of advancing this therapy to human clinical trials.

## Introduction

There is a high prevalence of immunoglobulin E (IgE)-mediated allergic diseases in industrialized countries involving over 1 in 3 people (1), with experts predicting that this may increase even further (2, 3). Allergen-specific immunotherapy (ASIT) was first performed by Noon in 1911 (4) and is still the gold standard for treating patients with allergic rhinitis, conjunctivitis, asthma, and allergies to hymenopteran venom type I allergies (2, 5). Unlike symptomatic allergy treatments, ASIT has a therapeutic effect by damping detrimental allergen-specific humoral and T-cell immune responses, moving them from Th2 dominant to more Th1 and T regulatory (Treg)-type responses (6). ASIT consists of administering gradually larger amounts of allergen to a patient in order to reduce symptoms arising from subsequent contact with the causative allergen (5).

For many years, ASIT has been administered by subcutaneous injection using soluble allergens in North America or allergen extracts adjuvanted with aluminum hydroxide or phosphate in Europe (5, 7). A full course of subcutaneous injection of ASIT provides a long-lasting therapeutic effect (2, 5) but is very protracted and involves many visits. It is also commonly associated with local and systemic adverse events (8).

Sublingual ASIT using allergens in the form of tablets or extracts is an alternative method licensed in some countries (9) and of increasing popularity. Its advantages are less side effects and a low risk of anaphylactic reactions (10, 11). Overall, sublingual ASIT has shown variable effectiveness when compared to the subcutaneous method (12). Sublingual ASIT requires higher allergen doses by 50–100-fold than subcutaneous ASIT, making it potentially more expensive (10). A major disadvantage of both subcutaneous and sublingual ASIT is the long course of therapy required that creates a compliance issue with many patients not completing a full course of therapy. The large number of injections in subcutaneous ASIT also increases the risk of encountering adverse effects (13).

Currently, several strategies to improve ASIT are under development, which can be divided into four categories: 1) changing the route of administration (intradermal and intra-lymphatic administration) (14, 15); 2) allergen modification (chemical modification of allergens and recombinant allergenic proteins or peptides) (16–18); 3) stimulating the innate immune response (using CpG agonists Toll-like receptor 9 (TLR-9) and tyrosine and monophosphoryl lipid A) (19, 20); 4) use of adjuvant and delivery systems [aluminum hydroxide or phosphate, probiotics, bacterial products, vitamin D, liposomes, virus-like particles, immunostimulatory complex (ISCOM), polymeric nanoparticles] (21). The quantity of allergen and number of doses can be significantly reduced when the vaccine is delivered with potent adjuvants, which positively impact safety of the ASIT (22).

Previously, allergic rhinitis from ragweed pollen was successfully addressed using a commercial product, Pollinex Quattro (Allergy Therapeutics, UK), which contains plant pollen extracts treated with glutaraldehyde, absorbed on L-tyrosine, and adjuvanted with monophosphoryl lipid A (MPL) injected four times 1 week apart (23), resulting in remission of symptoms for the duration of the allergy season (20). Wormwood pollen is among the top 10 global aeroallergens that cause allergic rhinitis and bronchial asthma (24). The goal of this pilot study, therefore, was to similarly test an ultrashort adjuvanted ASIT regimen for wormwood pollen-induced allergic bronchial asthma.

The native Art v 1 protein in natural pollen extract reacts with IgE in sera of >95% of patients with wormwood allergy (25). Recombinant Art v 1 produced in *E. coli* was used as the ASIT antigen in our study, as unlike natural pollen extract, it is less allergenic (it binds less to IgE) and may thereby cause less IgE-mediated mast cell activation when used, but nevertheless still be able to induce desensitization (26, 27). For this proof-of-concept study, we tested different allergens, adjuvants, and administration routes. In a mouse model, we evaluated the effectiveness of recombinant Art v 1 administered by intramuscular or subcutaneous route with Advax, Advax-CpG, ISA-51, aluminum hydroxide, or SWE adjuvant. We compared these to intranasal administration of Art v 1 protein entrapped in a chitosan-mannose nanoparticle.

## Materials and Methods

### Vaccine Formulation Preparation

The study used recombinant wormwood pollen Art v 1 protein (AtaGenix Laboratories, China) expressed in *Escherichia coli*, with purity >85% as determined by sodium dodecyl sulfate polyacrylamide gel electrophoresis (SDS-PAGE) and containing 129amino acids (AAs) with a molecular weight of 13 kDa. Adjuvants used included Advax and Advax-CpG (Vaxine Pty. Ltd., Australia), Alhydrogel^®^ 2% aluminum hydroxide (InvivoGen, CA, USA), Sepivac SWE™ (Seppic, France), Montanide ISA-51 (Seppic, France) (**Table 1**). Formulation was performed with increasing concentrations of Art v 1 protein (from 2 to 16 µg/dose) and adjuvants in a 50:50 ratio (by volume). The antigen–adjuvant mixture was vortexed for 30 s, aliquoted, and stored at 2°C–8°C until use. Emulsification of the antigen with ISA-51 adjuvant was performed according to the manufacturer’s instructions by syringe mixing cycles through the i-connector (20 slow and 40 fast stirring cycles).

**Table 1:** ASIT vaccine formulations.

Adjuvant	Art v 1 per 100 µl (dose) of vaccine, µg*	Dose/volume of adjuvant per 100 µl (dose) of vaccine	Route of administration
**Advax**	2, 4, 8, 16	1 mg	IM
**Advax-CpG**	2, 4, 8, 16	1 mg (Advax) + 10 µg (CpG)	IM
**Alum**	2, 4, 8, 16	1 mg	SC
**SWE**	2, 4, 8, 16	50 μl	IM
**ISA-51**	2, 4, 8, 16	50 μl	SC
**Nanovaccine**	4, 8, 16, 32	–	IN

^*^Antigen concentrations for first, second, third, and fourth immunizations, respectively.

IM, intramuscular; SC, subcutaneous; IN, intranasal.

To evaluate a nanovaccine for intranasal ASIT, mannose-conjugated chitosan nanoparticles were prepared by ionic gelation method as described previously (28). Briefly, to prepare mannose-conjugated chitosan (mCS), 200 mg of chitosan at 1% (weight/volume) (29) was slowly added to a mixture of mannose (Sigma) and sodium triacetoxyborohydride (Sigma) in 0.2 M borate buffer under magnetic stirring for 72 h at 56°C (30). The mCS was dialyzed 48 h against milli-Q-water and then lyophilized. Twenty milligrams was added to 20 ml of milli-Q water on a magnetic stirrer, the pH was adjusted to 4.3, and this was then mixed with 2 mg of Art v 1 protein in 3-(N-morpholino) propanesulfonic acid (MOPS) buffer at pH 7.4. Tripolyphosphate (Sigma) 5 mg in 10 ml milli-Q water was added dropwise, and the mCS–Art v 1 vaccine (nanovaccine) was obtained after centrifugation at 10,976 × g for 30 min, washed, dispersed in milli-Q water, and used for vaccination. The level of encapsulation of the antigens in the mCS was approximately 80%. This vaccine formulation was lyophilized and stored at 4°C until use.

### Mouse Sensitization

**Figure 1:**
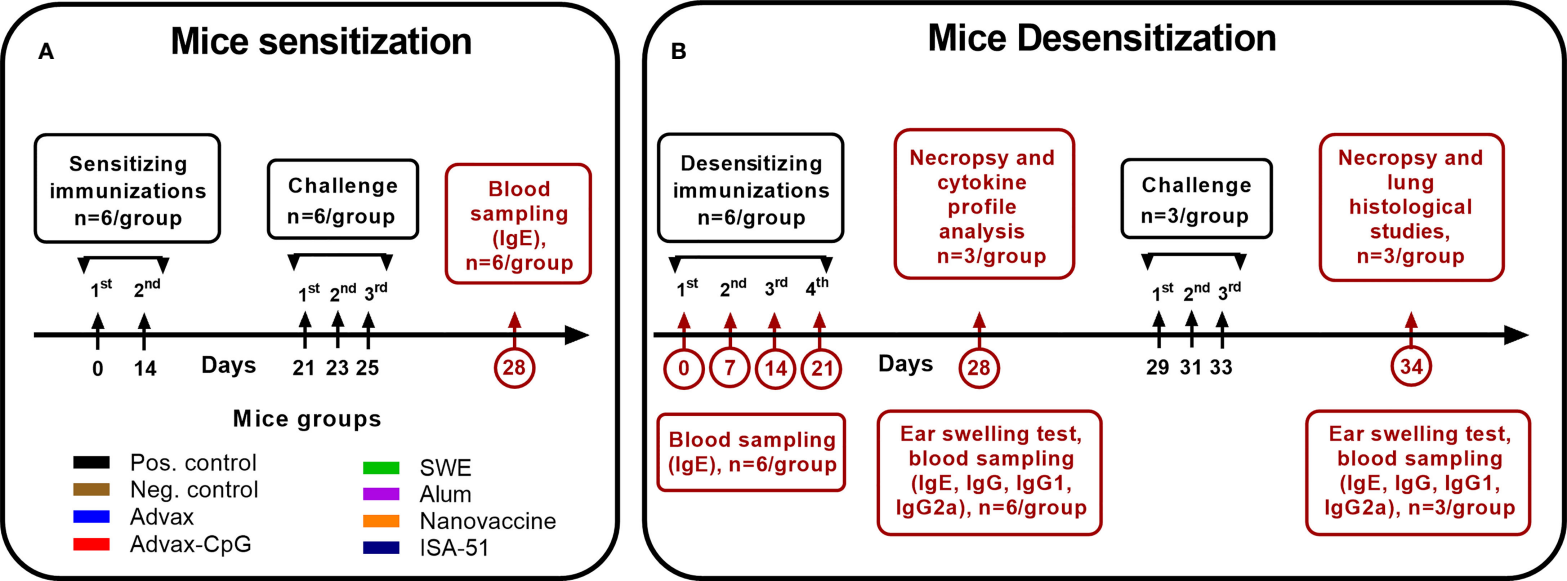
Experimental design. The scheme of sensitization **(A)** of mice by wormwood pollen extract and their desensitization **(B)** with vaccine formulations based on recombinant Art v 1 protein and various adjuvants, as well as wormwood pollen extract challenges, is depicted.

In this study, 8–12-week-old specific pathogen-free (SPF) male BALB/c mice (n = 6/group, 48 mice in total) were injected intraperitoneally twice at 14-day intervals with 1,000 PNU/200 µl of wormwood pollen extract (Burly, Almaty, Kazakhstan) adsorbed on aluminum hydroxide (InvivoGen; 1 mg/mouse). All mice were challenged three times on days 21, 23, and 25 by intranasal inoculation of wormwood pollen extract under ketamine-xylazine anesthesia at a dose of 200 PNU/20 µl or phosphate-buffered saline (PBS; negative control group). Blood samples were collected on day 28 to determine the level of total and Art v1-specific IgE. A graphical scheme of mouse sensitization with wormwood pollen extract is shown in **Figure 1A**.

### Determination of Total and Art v 1-Specific IgE by ELISA

Total IgE levels expressed in µg/ml were determined by ELISA MAX™ Standard Set Mouse IgE kit (BioLegend, USA) according to the manufacturer’s instructions.

For Art v 1-specific IgE detection, 0.5 µg/ml (100 μl/well) of recombinant Art v 1 protein was immobilized overnight on 96-well ELISA microplates using coating buffer (BioLegend). After washing, 200 µl/well ELISA Assay Diluent comprising PBS containing a blocking agent was added and incubated under constant shaking (300–330 rpm) on a PST-60HL thermal shaker (BIOSAN, Latvia) for 1 h at room temperature (RT). Plates were washed four times with wash buffer. Mouse serum samples were diluted 1:5 with ELISA Assay Diluent and 100 µl/well added and incubated for 1.5–2 h at RT with shaking. After washing (4×), anti-mouse biotinylated IgE detection antibody (1:200, BioLegend, 100 µl/well) was added and incubated for 1 h at RT with shaking. Plates were washed (4×) and incubated with horseradish peroxidase (HRP) streptavidin (BioLegend, 1:1,000, 100 µl/well) for 30 min at RT with shaking. After washing (5×), TMB substrate (BioLegend, 100 µl/well) was added. The color reaction was stopped by adding 2.5 M H_2_SO_4_ (100 µl/well), and the optical density (OD) was measured at wavelength 450 nm with reference wavelength 630 nm on a Stat Fax 2100 analyzer (Awareness Tech). The cutoff value for determining seropositivity was the average OD value of the negative sample + three times the standard deviation.

### Mouse Desensitization

To desensitize mice to wormwood pollen, they were immunized four times at 7-day intervals with the different vaccine formulations and routes of administration (**Table 1**). Mice from the positive (sensitized) and negative (non-sensitized) control groups were injected intramuscularly with PBS. On days 0, 7, 14, 21, and 28 of ASIT, blood samples were collected to evaluate the levels of Art v 1-specific and total IgE (n = 6/group). On day 28, the level of desensitization of the mice was assessed by the ear swelling test (n = 6/group). Additionally, we studied antigen-specific humoral (n = 6/group) and T-cell (n = 3/group) responses. A graphical scheme of the mouse desensitization regimen is shown in **Figure 1B**.

### Allergy Ear Swelling Test

In this study, 10 µl (100 PNU) of wormwood pollen extract was injected intradermally into the right auricle, with a negative control group being injected with PBS. After 1.5–2 h, the thickness of both auricles in each mouse was measured using an electronic digital micrometer MCC-25 DSWQ0-100II (China). Results are presented as the difference in thickness of the right (allergen injection) and left (no injection) auricles expressed in mm.

### Determination of Art v 1-Specific IgG and IgA Response

This assay was performed as described for the IgE ELISA. Briefly, Art v 1-specific IgG, IgG1, and IgG2a antibodies were determined in serially 2-fold diluted test samples with ELISA Assay Diluent buffer from a starting dilution of 1:125 to 1:4,096,000. To determine the presence of IgA antibodies, serum samples were diluted 1:5 with ELISA Assay Diluent. Anti-mouse biotinylated detection antibody for IgG (1:4,000, BioLegend), IgG1, IgG2a (1:1,000, BioLegend), and anti-IgA antibody (1:1,000, BioLegend) was used for detection. The cutoff value for IgG, IgG1, and IgG2a antibody titers was calculated based on the mean OD value of wells containing buffer only (blank) + three standard deviations.

### Assessment of Cellular Immune Response by Cytokine Profile

Mice (n = 3/group) were euthanized (cervical dislocation under ketamine/xylazine anesthesia). Harvested spleens were mechanically crushed into a single cell suspension using a cell strainer (Falcon^®^ 70-µm Cell Strainer) in a disposable sterile Petri dish (Piove di Sacco, Italy) using 10 ml of 3% fetal bovine serum (FBS; US Origin, Millipore Corp., Germany) in PBS. Erythrocytes in the suspension were then lysed with RBC lysis buffer (BioLegend). Splenocytes were cultured in a 5% CO_2_ incubator (INCO 153, Memmert, Germany) at 37°C in 24-well flat-bottomed plates (Sigma-Aldrich, USA) at a concentration of 1 × 10^6^ cells/well (1 ml) in RPMI-1640 + GlutaMax (Gibco) medium with 20 mM 4-(2-hydroxyethyl)-1-piperazineethanesulfonic acid (HEPES) (Gibco), 10% FBS (inactivated by heating), and 1% Antibiotic-Antimycotic (Gibco™) in the presence of 10 μg of purified recombinant protein Art v 1 (AtaGenix) or without protein (control without stimulation). Cells were incubated for 72 h, and the supernatant was then harvested and tested for interleukin (IL)-2, interferon (IFN)-γ, IL-12, IL-17A, tumor necrosis factor (TNF)-α, IL-4, IL-5, IL-6, IL-9, and IL-10 using ELISA MAX™ Deluxe Set Mouse (BioLegend) kits according to manufacturers’ instructions. The data were presented as the difference (delta) in cytokine concentrations in pg/ml between samples with and without protein stimulation.

### Assessment of the Allergen-Specific Immunotherapy Protective Efficacy After Challenge

Mice (n = 3/group) on day 29 after ASIT were subjected to allergen challenge on days 29, 31, and 33 by intranasal administration of wormwood pollen extract 200 PNU/20 µl under ketamine-xylazine anesthesia. For allergen inhalation, mice were transferred to a separate sealed 10-L chamber and sprayed with a FeelLife air pro 3 nebulizer (China) for 3 min with 1,000 PNU of wormwood pollen extract. Mice from the negative control group (n = 3) were treated with PBS. On day 34, blood samples were collected to determine IgE, IgG, IgG1, IgG2a, and IgA, ear swelling was measured, and then the mice were subjected to necropsy for histological analysis of the lungs for inflammatory reactions.

### Histological Analysis of Mouse Lungs

Mouse lungs were fixed in 10% buffered formaldehyde, washed in water, and treated with 4 portions of isopropyl 100% alcohol and two portions of xylene. Next, they were soaked in 4 portions of paraffin to make paraffin blocks that were then used for making 5-µm sections using a microprocessor-controlled microtome MZP-01 (KB Technom, Russia). The tissue sections were deparaffinized in 2 portions of xylene and 3 portions of ethyl alcohol with decreasing concentration (96%, 80%, 70%) and stained with hematoxylin (BioVitrum, Russia) and eosin (DiaPath, Italy). Following clarification in ascending ethyl alcohol concentrations (70%, 80%, 96%) and two portions of xylene, sections were covered with coverslips using Bio Mount synthetic medium (Bio Optica, Italy). The slides were observed in an Mshot microscope (China, model MF52-N), and photographs were taken at ×40 magnification using an Mshot MS23 camera in the Mshot Image Analysis System program. A ×1,000 magnification with an oil immersion lens was also used. A standardized scale was used for calibration, and all measurements were made in μm. Histologic examination of lungs was performed as previously described (31). Pathological changes were scored based on a histological scale as described (32, 33) (**Table 2**).

**Table 2:** Scale for assessing pathological changes in mouse lungs.

Evaluated trait	Features for evaluating the trait
**Inflammation including all pathological changes (Non-Type 2)**	
Perivascular/peribronchial inflammation	0 – no changes; 1 – moderate inflammation; 2 – pronounced inflammation; 3 – severe inflammation
Presence of neutrophils in foci of perivascular/peribronchial inflammation	0 – absent; 1 – less than 5 neutrophils per field with magnification ×1,000; 2 – more than 5 neutrophils per field with magnification ×1,000
Presence of eosinophils in foci of perivascular/peribronchial inflammation	0 – absent; 1 – single eosinophils on the field with magnification (×1,000); 2 – multiple eosinophils on the field with magnification (×1,000)
Metaplasia of the goblet cells in the bronchi	0 – absent; 1 – several goblet cells are present in one or two bronchioles; 2 – a large number of goblet cells are present in bronchioles
**Maximum score**	**16**
**Inflammation with pathological changes without neutrophils (Type 2)**	
Perivascular/peribronchial inflammation	0 – no changes; 1 – moderate inflammation; 2 – pronounced inflammation; 3 – severe inflammation
Presence of eosinophils in foci of perivascular/peribronchial inflammation	0 – absent; 1 – single eosinophils on the field with magnification ×1,000; 2 – multiple eosinophils on the field with magnification ×1,000
Metaplasia of the goblet cells in the bronchi	0 – absent; 1 – several goblet cells are present in one or two bronchioles; 2 – a large number of goblet cells are present in bronchioles
**Maximum score**	**12**

### Animal Housing and Bioethics

The studies in laboratory animals were performed in the M. Aikimbayev National Research Center for Especially Dangerous Infections of the Ministry of Health of the Republic of Kazakhstan. Microisolator technology in individually ventilated cages (Labproduct & Allentown, USA) were used to raise SPF animals.. Animals were provided with feed and water *ad libitum* with optimal environmental conditions: air temperature 20°C–24°C, humidity 45%–65%, illumination 325–350 Lx, noise level no more than 60 dB, air volume per animal 0.25 m^3^/h, airflow rate 0.2 m/s, number of animals per cage no more than 10, minimum cage area 180 cm^2^, full-fed food for adult animals 12 g/head/day, for young animals 5–8 g/head/day. Laboratory animals were provided with daily veterinary supervision. Autoclaved granulated feed, SSNIFF, standardized, enriched feed with vitamins, amino acids, and minerals (62 elements), with at least 19%–22% crude protein, without animal and growth supplements or antibiotics was used. Rehofix MK-2000 (JRS, Germany) was used as bedding material. Studies were conducted according to Protocol #3 dated June 16, 2020, approved by the Institutional Committee on the maintenance and use of laboratory animals of the M. Aikimbayev National Research Center for Especially Dangerous Infections.

### Statistical Analysis

GraphPad Prism 9.0.0 Software (San Diego, CA, USA) was used for plotting and statistical analysis of experimental data. Differences in antibody levels, cytokine production, ear swelling test results, and lung pathological changes between animal groups were assessed using Tukey’s multiple comparisons test or Šídák’s multiple comparisons test or Dunnett’s multiple comparisons test, as indicated. The detection limit of IgG titers and its isotypes was 7.0 log_2_. For the analysis of IgG, IgG1, IgG2a antibodies, geometric mean titers with 95% confidence intervals were calculated and expressed in log_2_. Evaluation of the interrelation of signs of allergic reactions in animals with various factors of humoral and cellular immune responses after both ASIT and challenge was assessed by multivariable Pearson correlation method. For all comparisons, P < 0.05 was considered a significant difference. All bars in the graphs represent the standard error of the mean.

## Results

### Total and Art v 1-Specific IgE in Sensitized Mice

**Figure 2:**
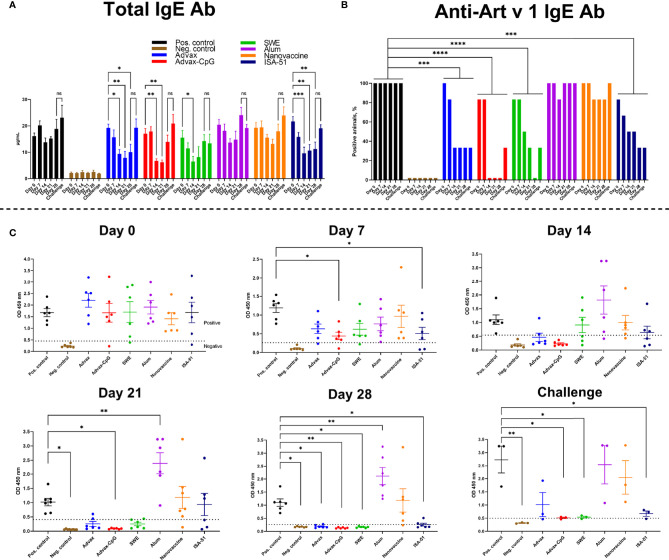
Reduction after ASIT of total **(A)** and Art v 1-specific **(B, C)** IgE in BALB/c mice sensitized with wormwood pollen. The number of mice positive for Art v 1-specific IgE on days (0, 7, 14, 21, 28) of ASIT and after challenge was estimated and expressed in percent. ASIT was performed with increasing doses of Art v 1 protein with Advax, Advax-CpG, SWE, aluminum hydroxide, or ISA-51. A group of mice that received intranasal Art v 1 protein-loaded mannose-chitosan nanoparticle ASIT was included for comparison. Challenges were performed by combined intranasal injection and inhalation of wormwood pollen extract. Differences in IgE levels between groups and ratio of animals seropositive for Art v 1-specific IgE were evaluated using Dunnett’s multiple comparisons test. A P value <0.05 was considered significant. *P < 0.05, **P < 0.01, ***P < 0.001, and ****P < 0.0001.

BALB/c mice were successfully sensitized to wormwood pollen extract as evidenced by a significant increase in both total and Art v 1-specific serum IgE in 83.3%–100% of sensitized animals compared to those in the negative control group (**Figures 2A–C**).

By day 14 of ASIT, there was a trend to reduced total serum IgE levels in almost all the vaccine groups, including the positive control group (without ASIT). However, only in the groups of mice that received ASIT with Advax, Advax-CpG, SWE, or ISA-51 adjuvants, the level of total IgE was significantly lower than before ASIT started (day 14 vs. day 0). Subsequent ASIT immunizations in the Advax and ISA-51 vaccine groups further reduced or maintained the already reduced levels of total serum IgE up to day 28. The number of mice positive for Art v 1-specific serum IgE in the Advax-, Advax-CpG-, SWE-, and ISA-51-adjuvanted vaccine groups decreased with each ASIT immunization and was significantly lower than that in the positive control group across the entire observation period **(** **Figure 2B** **)**. Notably, 100% of the Advax-CpG group were seronegative to Art v 1-specific IgE after two injections (day 14), while the SWE group required four injections to achieve the same result (on day 28). Although 33.3% (2/6) of the Advax and ISA-51 groups remained positive for Art v 1-specific IgE **(** **Figure 2B** **)**, these levels were significantly lower than that in the positive control group **(** **Figure 2C** **).** In the Alum+Art v 1 and intranasal nanovaccine groups, there was no significant decrease in either total or Art v 1-specific IgE during the ASIT treatment. Indeed, in the Alum+Art v 1 group, there was an increase in Art v 1-specific IgE on days 21 and 28 of ASIT.

A triple challenge with wormwood extract slightly increased (vs. 28 days of ASIT) the level of total and Art v 1-specific IgE in all experimental groups, but the levels in Advax-CpG, SWE, and ISA-51 groups remained significantly lower than that in the positive control group. After triple challenge, the number of seropositive animals in the Advax-CpG and SWE vaccine groups increased from 0% to 33.3% (1/3), while in the Advax and ISA-51 groups, it remained at the same level (33.3%).

### Antibody Response Analysis After Allergen-Specific Immunotherapy and Challenge

**Figure 3:**
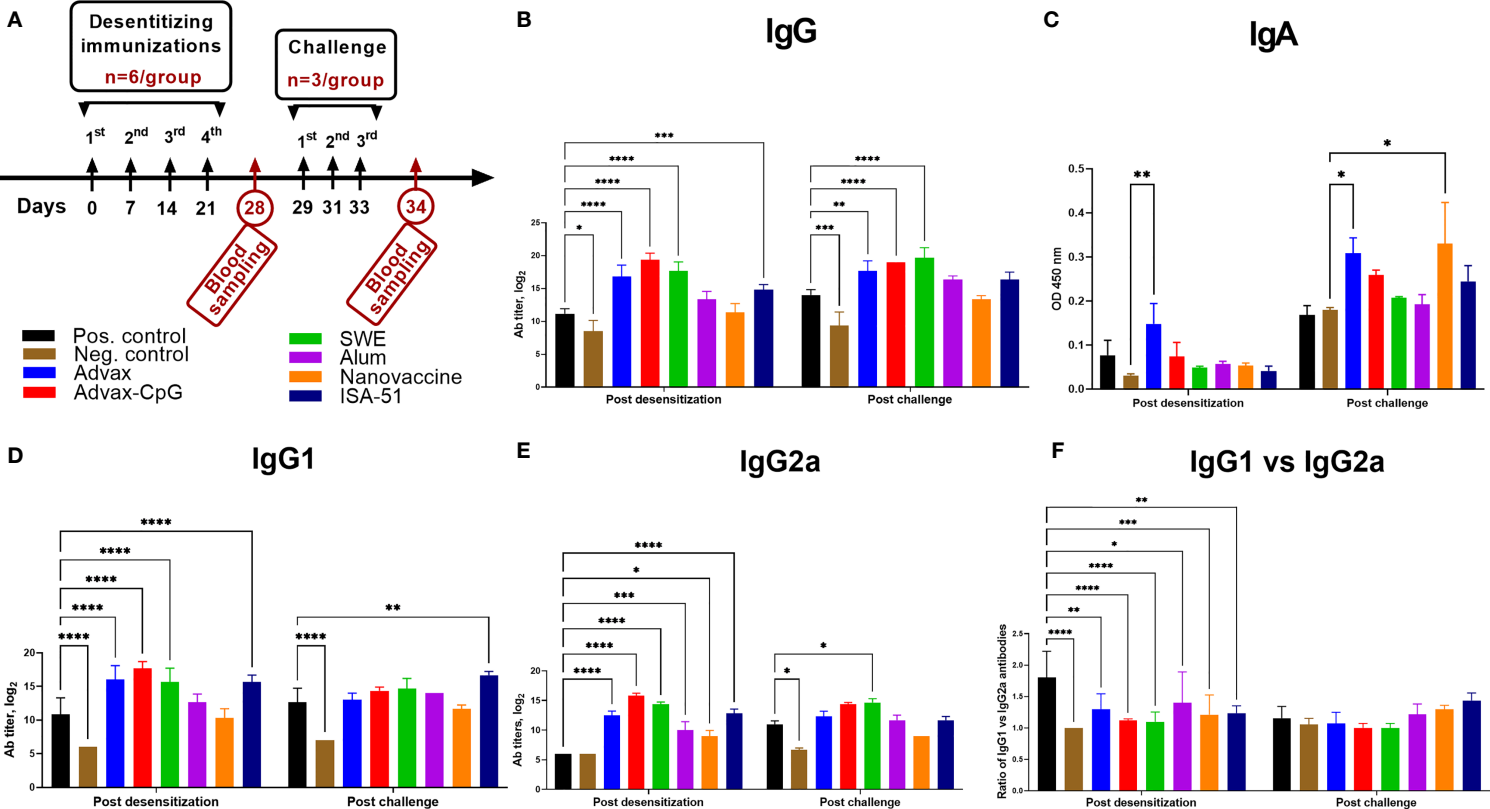
Art v 1-specific serum IgG **(B)**, IgG1 **(D)**, IgG2a I **(E)**, IgA **(C)**, and IgG1 to IgG2a ratio **(F)** in BALB/c mice after ASIT and challenge. ASIT was performed with Art v 1 protein formulated with Advax, Advax-CpG, SWE, aluminum hydroxide, or ISA-51 **(A**, study design). A group of mice that received intranasal Art v 1 protein-loaded mannose-chitosan nanoparticle ASIT was included for comparison. Challenge of animals was performed by intranasal injection and inhalation of wormwood pollen extract. Antibody levels are presented as geometric mean titers with 95% confidence intervals and expressed in log_2_. Differences in antibody levels between groups were assessed using Šídák’s multiple comparisons test. A P < 0.05 value was considered significant. *P < 0.05, **P < 0.01, ***P < 0.001, and ****P < 0.0001.

ASIT with all vaccine formulations except for the alum and nanovaccine groups resulted in a significant increase in Art v 1-specific IgG, IgG1, and IgG2a when compared to the positive control group (**Figures 3A, B, D, E** **)**. In the positive control group, there was a pronounced polarization toward Art v 1-specific IgG1 (Th2 immune response) (**Figure 3F**). Notably, only in the Advax group was there a significant increase of Art v 1-specific serum IgA after ASIT when compared to that of the negative control group (**Figure 3C**). After allergen challenge, Art v 1-specific IgA was also significantly increased in the nanovaccine group. Allergen challenge induced distinctive changes in Art v 1-specific IgG, IgG1, and IgG2a levels. A significant increase in Art v 1-specific IgG after challenge compared to those after ASIT (P = 0.007–0.01) was observed only in the Alum and positive control groups. By contrast, in the Advax, Advax-CpG, and SWE groups, Art v 1-specific IgG after challenge remained unchanged. In the positive control group, the ratio of Art v 1-specific IgG1 to IgG2a decreased after challenge due to a significant increase in Art v 1-specific IgG2a. In the ISA-51 group, there was significant increase in Art v 1-specific IgG1 **(** **Figure 3D** **)**, with an increase in the ratio of IgG1 to IgG2a **(** **Figure 3F** **)**. A post challenge increase in Art v 1-specific IgG2a was observed only in the SWE group (**Figure 3E**).

### Cytokine Profile Analysis After Allergen-Specific Immunotherapy

**Figure 4:**
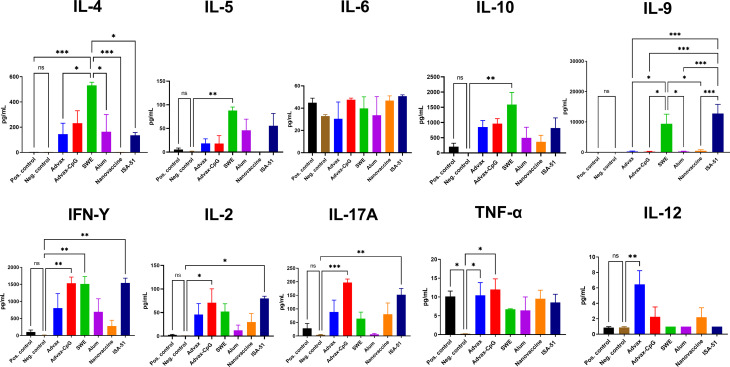
Production of Th1, Th2, and Th17 cytokines in splenocyte suspensions after ASIT. Cytokine data are presented as the difference (delta) in cytokine concentrations between samples with or without stimulation with Art v 1. Differences between groups were assessed using Tukey’s multiple comparisons test. NS, Non significant. P < 0.05 value was considered a significant difference. *P < 0.05, **P < 0.01, ***P < 0.001.

In sensitized mice after ASIT, depending on the vaccine formulation, a diverse splenocyte Art v 1-specific cytokine response was observed (**Figure 4**). Only the SWE group showed production of both IFN-γ and IL-4, indicators of Th1 and Th2 immune responses, respectively. IL-4 production in this group was much higher than all other vaccine groups. This group showed increased production of other Th2 cytokines such as IL-5 and IL-9. Notably, only in the SWE group was there a significant production of IL-10, a Treg cytokine. In the ISA-51 vaccine group, an opposite result was observed with predominantly Th1 and Th17 cytokines, IFN-γ, IL-2, and IL-17A. A common feature to the two oil adjuvant formulations, SWE and ISA-51, was induction of IL-9. Advax-CpG elicited exclusively Th1 (IFN-γ, IL-2) and other non-Th2 cytokines (IL-17A and TNF-α). Advax induced IL-12, a Th1 cytokine. The Alum+Art v 1 and nanovaccine did not induce any cytokines. The sensitized positive control group only produced higher TNF-α when compared to that of the negative control group.

### Evaluation of the Effectiveness of Allergen-Specific Immunotherapy With Different Vaccine Formulations

**Figure 5:**
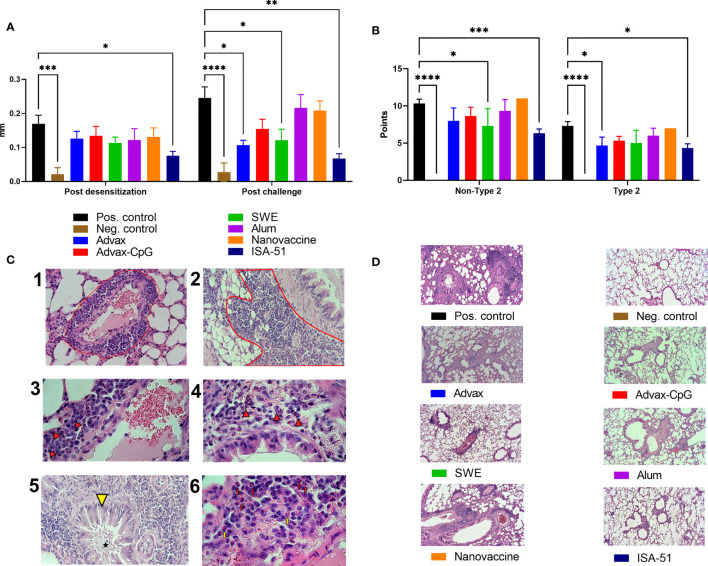
Efficacy of ASIT in BALB/c mice as assessed by ear swelling test **(A)** and histological analysis of the lungs **(B, D)**. The ear swelling test was performed after ASIT and challenge and the analysis of pathological changes in the lungs of mice after intranasal challenge with wormwood pollen extract. The results of the ear swelling test are presented as the difference in the thickness of the mouse auricles with and without the allergen/PBS injection expressed in mm. Histological analysis of lung samples after challenge was performed according to two scales: all pathological changes (maximum 16 points, Non-Type 2 inflammation) and in the absence of neutrophils (maximum 12 points; classic allergic Type 2 inflammation). We present representative pictures of the lungs from different groups at a magnification ×100 **(C)**. –1 - Perivascular inflammation (red outline, ×400); –2 - Peribronchial inflammation (red outline, ×400); –3 - neutrophils (arrows) as part of the perivascular inflammation, ×1,000; –4 - Neutrophils (arrows) as part of the peribronchial inflammation, ×1,000; –5 - Metaplasia of goblet cells (yellow arrowhead) and mucus accumulation (asterisk) in the bronchiolar cavity, ×400; –6 - Neutrophils (red arrows) and single eosinophils (yellow arrows) in the perivascular inflammatory infiltrate, ×1,000. Differences in studied parameters between groups were assessed using Šídák’s multiple comparisons test. P < 0.05 value was considered significant. *P < 0.05, **P < 0.01, ***P < 0.001, and ****P < 0.0001.

Clinically, the efficacy of ASIT in mice was evaluated based on the ear swelling test and pathological changes in the lungs. Post-desensitization, only ISA-51-adjuvanted vaccine caused a significant reduction in auricle thickening in response to allergen challenge compared to the positive control group (**Figure 5A**). After the third challenge, there was significantly less auricle thickening in Advax and SWE groups as well as the ISA-51 group compared to that in the positive control group.

Histological analysis of the lungs after challenge was based on the scores of perivascular and peribronchial inflammation (with or without eosinophils and neutrophils in inflammation foci), as well as metaplasia of goblet cells in the bronchi (**Figure 5B**). No classic signs of bronchial asthma (hyperplasia and hypertrophy of smooth muscles, inflammatory infiltrates in peribronchial and perivascular areas containing eosinophils) of Type 2 were found in mice, except metaplasia of goblet cells and mucus production in bronchioles (**Figure 5C**). Neutrophils rather than eosinophils were increased in the lung infiltrates (**Figure 5C**), indicating predominantly non-Type 2 inflammation. The results showed that all vaccine groups after challenge had pathological changes of both types of inflammation in the lungs, but only in the ISA-51 group were changes significantly lower than those in the positive control group. The SWE group also showed significant (vs. positive group) protection from pathological lung changes. Significant protection against Type 2 inflammation was seen in the Advax group. The highest level of pathological changes with both types of inflammation was seen in the lungs of the positive control, nanovaccine, and alum-adjuvanted groups (**Figures 5B, D** **)**. They were found to have moderate peribronchial inflammation with few neutrophils and eosinophils present in the foci of inflammation. Metaplasia of goblet cells was observed in most bronchioles with pronounced perivascular inflammation. Many neutrophils and eosinophils were present in the foci.

**Figure 6:**
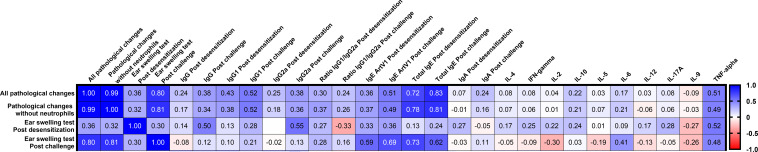
Correlation matrix analysis between allergy responses (lung pathology and ear swelling) and Art v 1 allergen-stimulated humoral or T-cell responses after ASIT and challenge. The color refers to the r value scale (-1 to 1) shown on the right. The number in each cell represents the actual r value. The analysis was performed using the Pearson multivariate correlation method.

Furthermore, we evaluated the correlation of allergic reactions in animals with respect to various humoral and cellular parameters after ASIT and challenge (**Figure 6**). Both types of lung inflammation were significantly positively correlated with the amount of ear swelling after pretreatment challenge (r = 0.8–0.81, P < 0.0001) but were not significantly different after ASIT (r = 0.32–0.36, P = 0.12–0.08). Interestingly, lung pathological changes were better correlated with total serum IgE levels after challenge (r = 0.81–0.83, P < 0.0001) than Art v 1-specific IgE levels (r = 0.49–0.51, P = 0.014–0.011). No significant correlations between lung inflammation and Art v 1-specific IgG, IgG1, IgG2a, or IgA antibodies or cytokine responses were found apart from levels of allergen-stimulated TNF-α that correlated with lung pathology after challenge (r = 0.49–0.51, P = 0.014–0.011).

## Discussion

The increase in prevalence of allergic diseases globally and issues with current ASIT including compliance and tolerability necessitate the development of new and improved ASIT. The objective of this study was to test various adjuvanted ASIT vaccines to assess their ability to alleviate bronchial asthma caused by wormwood pollen allergy. The effectiveness of such an approach was previously demonstrated for treatment of ragweed pollen allergic rhinitis (20, 23). The aim of ASIT is to induce a favorable shift toward Treg/Th1-type responses with parallel induction of IgE-blocking IgG antibodies. Various adjuvants have been tested in ASIT. Allergen adsorbed with aluminum adjuvant has been used in various studies (34). However, aluminum adjuvants have serious drawbacks, as they strongly stimulate Th2 responses and can exacerbate IgE and eosinophilia, which may aggravate the risk of adverse reactions (35). Consequently, the search for new adjuvants for use in ASIT that are capable of inducing allergen tolerance while avoiding the induction of excess Th2 responses is warranted. Among the many new adjuvants, we chose ones that had previously been well investigated. Advax™ adjuvant (Vaxine Pty. Ltd., Australia) is a polysaccharide particle (delta inulin) derived from polyfructofuranosyl-D-glucose. Previously, this adjuvant was widely studied in many vaccines (avian influenza H5N1, seasonal influenza, hepatitis B) (36, 37) with more than 10 human clinical trials involving over 2,000 volunteers. Advax™ adjuvant has been shown to induce both Th1 and Th2 cell responses, enhancing the production of IgG without increasing IgE (38). Advax adjuvant has already been tested successfully in preclinical (39) and clinical studies (40) of ASIT against bee and ant venom allergy. Advax can be used in combination with a CpG oligonucleotide (Advax-CpG) that stimulates TLR-9, which results in even stronger activation of humoral and cellular immunity (41). Advax-CpG adjuvant efficacy has been demonstrated to be effective in vaccines against tuberculosis (41, 42), Alzheimer’s disease (43), and coronavirus disease 2019 (COVID)-19 (44), among others. This study also tested the SWE squalene emulsion adjuvant that mimics the well-known MF59 adjuvant (Sequirus, USA) included in commercial influenza vaccines (45, 46). We recently tested SWE adjuvant in a candidate COVID-19 vaccine in hamsters where it showed promising immunogenicity and protection (47). Montanide ISA-51 VG is a “water in oil” (W/O) emulsion, consisting of mineral oil and a surfactant from the mannide monooleate family (48). This adjuvant has been tested in thousands of people in clinical trials of cancer, human immunodeficiency virus (HIV)/acquired immunodeficiency syndrome (AIDS), and malaria (49) vaccines. A therapeutic lung cancer vaccine containing ISA-51 adjuvant is licensed in Cuba (50), and it has also been used in influenza vaccine trials (51, 52). We also evaluated the intranasal method of ASIT delivery. The advantages are it requires a low dose of vaccine and provides a large absorption area (53). The mannose surface-labeled chitosan nanoparticle-entrapped vaccine antigens delivered to mucosal sites protect the vaccine cargo from degradation, increase the stability, and ensure targeted delivery to antigen-presenting cells (APCs) (54, 55). Chitosan is a biocompatible and bioavailable natural polymer, and its positively charged amino groups electrostatically interact with the negatively charged sialic acid of mucus and epithelial surfaces, making it a strong mucoadhesive vehicle (56). By inclusion of a calcium-dependent (type C) mannose receptor of the lectin family on chitosan nanoparticle, it binds to dendritic cells and macrophages (57), resulting in adjuvant effects (58, 59). This approach has been tested in vaccines against swine influenza (58) and avian salmonellosis (59).

This study confirmed the lack of efficacy of an ultrashort ASIT regimen using aluminum hydroxide adjuvant, which actually increased the levels of total and Art v 1-specific IgE with no protection to aeroallergen challenge. These data on alum are consistent with other reports (35), with alum-adsorbed allergen only effective in long-term ASIT regimens involving large numbers of injections. Our intranasal nanovaccine similarly showed no efficacy despite it increasing Art v 1-specific IgA in the serum after challenge.

In contrast, recombinant Art v 1 formulated with Advax, Advax-CpG, SWE, or ISA-51 provided encouraging results, significantly decreasing both total and Art v 1-specific IgE with concurrent increase in IgG isotypes when compared to the sensitized positive control group. In the cytokine profile, there was a shift to a Th1 (Advax-CpG, ISA-51, and Advax) or a balanced Th1/Th2 (SWE) type immune response. Unexpectedly, Advax-CpG, despite inducing IgG and a Th1 switch with associated suppression of Art v 1-specific IgE, did not have a major effect on clinical protection against challenge. This may be related to increased TNF-α production that positively correlated with lung pathology after challenge. TNF-α has been shown to be involved in the development of asthma, chronic bronchitis, chronic obstructive pulmonary disease, acute lung injury, and acute respiratory distress syndrome (60). Alternatively, this finding may reflect IL-9, which was increased in the oil emulsion adjuvant groups. IL-9 plays a key role in the induction of Th17 cells and in resolution of inflammation *via* Tregs (61). Hence, the lack of IL-9 induction in the Advax-CpG group may indicate a lack of Treg induction. While the lack of Treg induction may be beneficial in a typical vaccine against infectious disease, it may be less conducive to allergy vaccines, where induction of high levels of Treg is desired.

Although Advax adjuvant showed significant production of TNF-α, we observed protection against Type 2 inflammation in the lungs in allergen-challenged animals possibly due to substantial production of the Th1 cytokine, IL-12. Because it favors Th1, but not Th2-type cells and IgE, Advax may help regulate IgE-associated inflammation in allergic lung disease (62). Interestingly, Advax was the only adjuvant that resulted in increased serum Art v 1-specific IgA after challenge, which might have contributed to lung protection from inflammation, as IgA can play a potent anti-inflammatory role *via* interaction with FcαRI and DC-SIGN/SIGNR (63).

Paradoxically, ASIT with SWE adjuvant preferentially induced Th2 bias cytokines (IL-4, IL-5) but was associated with a significant decrease in total and Art v 1-specific IgE. This IgE reduction may have been due to suppressive effects of IL-10 or IL-9-associated activation of Tregs. However, the level of allergic reaction after ASIT in terms of ear swelling did not decrease compared to the positive control group, although lung inflammation did reduce.

Challenge of mice with wormwood pollen extracts caused both typical allergic inflammation (Type 2) and non-typical (Non-Type 2) pathological changes in the lungs, which is common in bronchial asthma in rodent models (64). On this basis, we used two types of scoring to evaluate the efficacy of vaccine formulations, taking into account Non-Type 2 (all pathological changes in the lungs) and Type 2 (pathological changes without neutrophils) inflammation. Notably, only the ISA-51-adjuvanted vaccine formulation provided protection against both types of lung inflammation after challenge and had significantly less ear swelling. Further investigations are required to understand this mechanistically. Interestingly, in terms of immune response profile, the ISA-51-based vaccine formulation was not significantly different from the Advax-CpG group, inducing comparable levels of Th1 cytokines (IFN-γ, IL-2) and both reducing IgE levels. However, only ISA-51 was associated with increased IL-9, which may thereby represent the key to understanding the reduction in lung inflammation by the ISA-51-adjuvanted vaccine formulation. One downside to ISA-51-adjuvanted vaccines is that they are very viscous and we encountered difficulties in formulating the preparation using a syringe connector due to high viscosity of the resulting emulsion.

A key goal of allergy research is to identify correlates of protection that could be used to predict the effectiveness of ASIT. Our analysis showed that lung inflammation in mice correlated strongly with the level of ear swelling data after challenge, but not after ASIT. We also found that clinical protection of mice from pollen allergy best correlated with the level of total serum IgE rather than the level of Art v 1-specific serum IgE. Pollen allergens, including weeds, release not only allergens but also pro-inflammatory and immunomodulatory lipids and adenosine, which act as critical cofactors in the development of allergic lung inflammation (65). In this case, both specific and nonspecific IgE may contribute to allergy severity.

Limitations of this study included the limited group sizes used, together with the fact that the study has yet to be repeated. There were no antigen specificity controls, whereas experiments with adjuvant and an irrelevant antigen are needed as well as reciprocal experiments to show the effect of the immunotherapy with Art v 1 and adjuvant on responses to a non-cross-reacting antigen. In the present studies, we used wormwood pollen extract as the antigen (in 200, 400, 800, and 1,600 PNU/mouse doses on days 0, 7, 14, and 21 of ASIT, respectively) in all vaccine formulations (except nanovaccine) tested for comparison. However, we did not see any improvement that we put down to use of a wormwood pollen allergoid. In the ultrashort regimen of ASIT, the control antigen (wild pollen extract) with all vaccine formulations showed the worst protection against inflammatory reactions in the lungs of mice after challenge compared to Art v 1 antigen. It is not possible to know whether the mouse model would recapitulate human wormwood pollen allergy. In particular, mice have significant differences to human immune responses, and this may influence study outcomes. Nevertheless, the study clearly shows the importance of adjuvants to ASIT outcomes and highlights the way in which adjuvants can be used to differentially shape the immune response. It further highlights the need for more detailed mechanistic studies into how adjuvants might have beneficial clinical effects on ASIT.

Future studies will evaluate the safety and efficacy of a wormwood pollen ASIT vaccine using ISA-51 or Advax adjuvants in order to try and determine a final ASIT formulation for advancement into human clinical trials.

## Data Availability Statement

The original contributions presented in the study are included in the article/supplementary material. Further inquiries can be directed to the corresponding author.

## Ethics Statement

Studies with laboratory mice were conducted according to Protocol #3 dated June 16, 2020, approved by the Institutional Committee on the maintenance and use of laboratory animals of the M. Aikimbayev National Research Center for Especially Dangerous Infections.

## Author Contributions

Conceptualization: KaissarT, NP, TaN. Data curation: KairatT. Formal analysis: KaissarT, NP, TaN. Funding acquisition: KaissarT. Investigation: KairatT, MB, GF, TeN, SR. Methodology: KaissarT, MB. Project administration: KaissarT. Resources: KaissarT. Software: KaissarT. Supervision: KaissarT. Validation: KairatT. Visualization: KaissarT, NP, GJR, MB. Writing—original draft: KaissarT . Writing—review and editing: KaissarT, NP, GJR. All authors contributed to the article and approved the submitted version.

## Funding

This research was funded by the Science Committee of Ministry of Education and Science of the Republic of Kazakhstan (Grant No. AP08051924). Studies to evaluate vaccine formulations based on SWE and ISA-51 adjuvants were funded by the T&TvaX. The funder was not involved in the study design, collection, analysis, interpretation of data, the writing of this article or the decision to submit it for publication. Development of Advax and Advax-CpG adjuvant was supported by funding from the National Institute of Allergy and Infectious Diseases of the National Institutes of Health under Contract HHSN272201400053C and HHSN272201800044C.

## Conflict of Interest

KaissarT and KairatT are affiliated with T&TvaX. NP is affiliated with Vaxine, a company holding rights to Advax and Advax-CpG adjuvants.

The remaining authors declare that the research was conducted in the absence of any commercial or financial relationships that could be construed as a potential conflict of interest.

## Publisher’s Note

All claims expressed in this article are solely those of the authors and do not necessarily represent those of their affiliated organizations, or those of the publisher, the editors and the reviewers. Any product that may be evaluated in this article, or claim that may be made by its manufacturer, is not guaranteed or endorsed by the publisher.
